# Prediction Model of Immunosuppressive Medication Non-adherence for Renal Transplant Patients Based on Machine Learning Technology

**DOI:** 10.3389/fmed.2022.796424

**Published:** 2022-02-18

**Authors:** Xiao Zhu, Bo Peng, QiFeng Yi, Jia Liu, Jin Yan

**Affiliations:** ^1^Nursing School of Central South University, Changsha, China; ^2^Nursing Department of Third Xiangya Hospital of Central South University, Changsha, China; ^3^Research Center of Chinese Health Ministry on Transplantation Medicine Engineering and Technology, The Third Xiangya Hospital, Central South University, Changsha, China

**Keywords:** immunosuppressive medication, non-adherence, prediction model, renal transplant patients, machine learning technology

## Abstract

**Objectives:**

Predicting adherence to immunosuppressive medication (IM) is important to improve and design future prospective, personalized interventions in Chinese renal transplant patients (RTPs).

**Methods:**

A retrospective, multicenter, cross-sectional study was performed in 1,191 RTPs from October 2020 to February 2021 in China. The BAASIS was used as the standard to determine the adherence of the patients. Variables of the combined theory, including the general data, the HBM, the TPB, the BMQ, the PSSS and the GSES, were used to build the models. The machine learning (ML) models included LR, RF, MLP, SVM, and XG Boost. The SHAP method was used to evaluate the contribution of predictors to predicting the risk of IM non-adherence in RTPs.

**Results:**

The IM non-adherence rate in the derivation cohort was 38.5%. Ten predictors were screened to build the model based on the database. The SVM model performed better among the five models, with sensitivity of 0.59, specificity of 0.73, and average AUC of 0.75. The SHAP analysis showed that age, marital status, HBM-perceived barriers, use pill box after transplantation, and PSSS-family support were the most important predictors in the prediction model. All of the models had good performance validated by external data.

**Conclusions:**

The IM non-adherence rate of RTPs was high, and it is important to improve IM adherence. The model developed by ML technology could identify high-risk patients and provide a basis for the development of relevant improvement measures.

## Introduction

Over past decades, with improved immunosuppressive therapy and surgical techniques, improvements in graft survival have been achieved in the early-post transplantation phase (1–3). However, successful long-term kidney graft outcomes remain suboptimal. The therapeutic regimens of renal transplant patients (RTPs) typically involve taking various prescribed medications per day, including immunosuppressive medication (IM). They need to receive immunosuppressant therapy for as long as their grafts continue to function. Successful long-term kidney graft outcomes remain suboptimal, with IM non-adherence considered as an important contributing factor. Nevertheless, non-adherence is common, occurring in 23.21–44.2% of Chinese renal transplant recipients in our previous studies (4–6). IM non-adherence is a major issue among transplant recipients that can lead to misdiagnosis, rejection, graft loss or death.

Recently, a few studies have explored the risk prediction of medication non-adherence in this field. Several theories/models have been formulated to help predicting and understanding medication adherence. Our previous studies illustrated that perceived seriousness and barriers were closely associated with immunosuppressive adherence, utilized the Health Belief Model (6). Attention is attracted by potentially modifiable factors, such as social support, experiences on dialysis, side effects, features of the treatment regimen, intentions and beliefs, forgetfulness and mental health issues, playing greater roles than other factors in the development of medication non-adherence of renal transplant receipts (7). Nevertheless, few models were well-suited to identifying all of the factors that contribute to non-adherence to a prescribed medical regimen as crucial as immunosuppressive therapy, and each has limitations. For example, the theory of planned behavior (TPB), a successful psychosocial-cognitive model for predicting a wide range of health-related behaviors, has been proposed to add variables, such as past behavior, to enhance the prediction (8). Therefore, we favor a combined model based on our previous studies to reflect the multilevel approach to medication non-adherence. A combined theory model with medication non-adherence has received little attention in transplantation.

Machine learning (ML), which itself is a subset of a broader universe of computational learning in artificial intelligence, is now embedded in many aspects of health care processes, including biomedical research and health care delivery (9, 10). There were already some good examples of using ML technology to build accurate prediction models in the medical field. Compared to traditional statistical methods, ML has more advantages in the ability to identify variables related to clinical outcomes, to predict performance, to manage complex relationships between variables and to process big data (11–13). Prediction models for nephropathy and renal transplantation outcomes based on ML are rapidly emerging. Such models, if adequately reported, could guide treatment decision-making, predict adverse outcomes, and streamline perioperative health care management (14–16). The application of ML technology in medicine behavior monitoring is promising, and it could help us to better understand the complexity of behaviors and intentions related to IM adherence.

This study aimed to examine the correlation between variables of the combined theory and non-adherence behavior in RTPs. In particular, ML techniques were used to build the models and identify the variables most relevant to non-adherence. The results provided predictive models that using clinically available variables to identify at-risk patients and find potential directions for interventions.

## Methods

### Study Population

**Figure 1:**
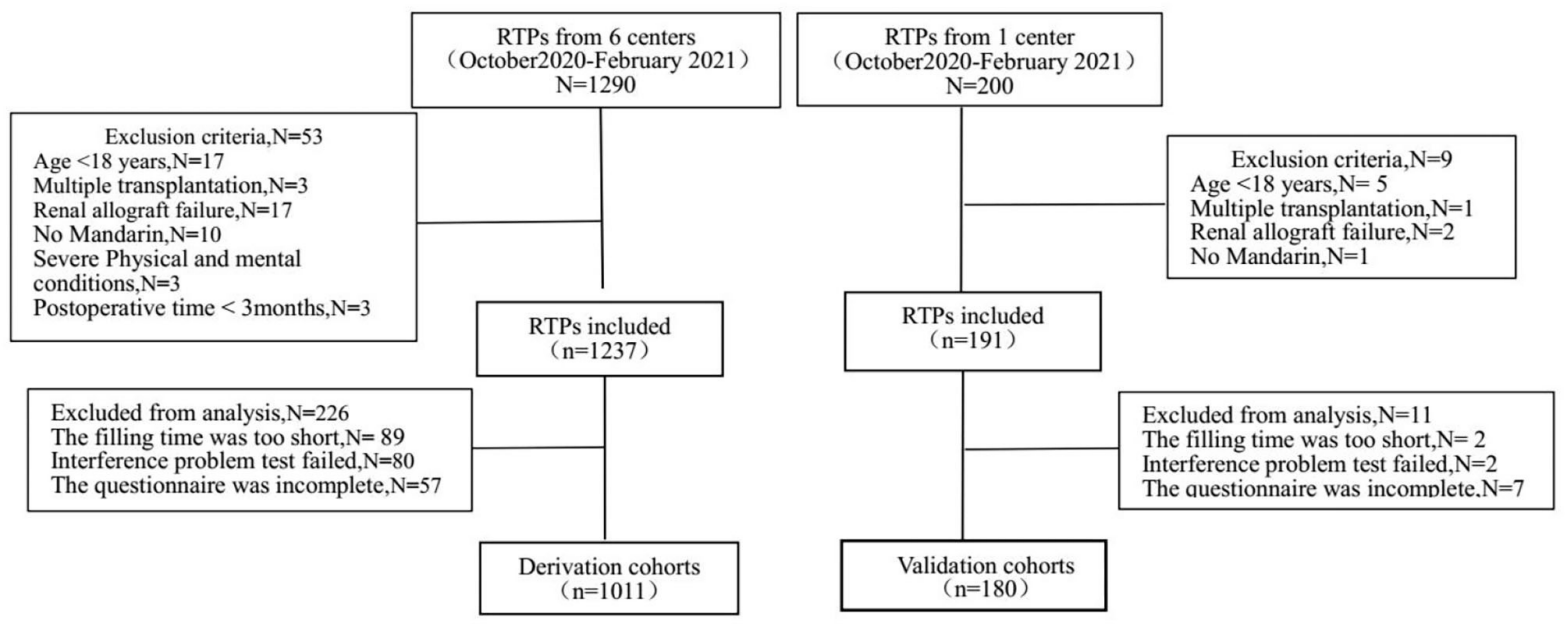
The enrollment of patients in the derivation cohort and validation cohort.

Renal transplant recipients attending the transplantation follow-up outpatient clinic at the Third Xiangya Hospital (Changsha, Hunan Province, China) and five other transplantation outpatient clinics (Chenzhou First People's Hospital, the Second Xiangya Hospital, Yueyang First People's Hospital, the Second Affiliated Hospital of South China Medical University and Yiyang Central Hospital, Hunan Province, China) had postsurgery times of at least 3 months. The enrollment of RTPs in the derivation cohort and validation cohort is shown in Figure 1. Finally, 1,011 patients were enrolled in the derivation cohort, and 180 patients were enrolled in the validation cohort between October 2020 and February 2021. This study followed the tenets of the Declaration of Helsinki and was approved by the Ethics Committee of the Third Xiangya Hospital (2019-SS161), Changsha, China. Written informed consent was obtained from all of the study participants.

### Study Design

When patients came for follow-up visits, physicians in the outpatient clinic invited them to participate in this study. Interested patients would guide by a research nurse. Patients who met the inclusion criteria received an informed consent form during the consultation with the nurse. In order to improve participants' enthusiasm in filling out the questionnaire, patients were given a free copy of the book *Kidney Transplant Patient Management Manual* in appreciation of their participation after completing the questionnaire.

### Questionnaire Quality Control

We controlled the quality of questionnaire collection from three aspects as shown in the Figure 1.

(1) First of all, we selected patients strictly according to the inclusion and exclusion criteria. In derivation cohort, we recruited 1,237 patients who met the inclusion criteria, and excluded 53 patients who did not meet the inclusion criteria (such as age < 18 year, multiple transplantation). (2) Secondly, we controlled the questionnaire filling process strictly. In derivation cohort set, we collected questionnaires from 1,237 patients. Before the formal study, we invited two postoperative patients, one medical student and one preoperative patient to complete the questionnaire independently, with an average completion time of 6 min and 48 s. Those questionnaires that took <25% of the average completion time were rejected (*N* = 89). Interference questions were added to the scale TPB and PSSS, and the questionnaires with contradictory answers would be removed (*N* = 80). At the same time, the outpatient doctors and nurses answered the questions during the questionnaire filling process. (3) Finally, we carefully checked whether the questionnaire was filled in completely, and deleted incomplete questionnaires (*N* = 57) in derivation cohort. The collection of questionnaires in the validation cohort followed the same process.

### Instruments

In our study, a total of seven questionnaires were included.

### General Data Questionnaire

Participants' demographic characteristics were collected from follow-up data (follow-up system in each center), including age, sex, body mass index (BMI), marital status, work, religion, education, household income, preoperative drinking history, time after transplantation and organ source. Supplementary Information including past behavior about taking medicines and adverse reactions during the medication period was also collected.

### Basel Assessment of Adherence to Immunosuppressive Medications Scale

BAASIS is a self-report questionnaire developed by the Leuven-Basel Adherence Research Group (17). We examined two dimensions of IM non-adherence in the questionnaire in the past 4 weeks: implementation and discontinuation. Implementation was assessed by four questions (dose taking, drug holidays, timing deviation more than 2 h from the prescribed time, and dose reduction). Discontinuation was assessed by one question (completely stopping medication intake). Any question option that was not “No” or “None” was determined as non-adherence. The scale was translated into Chinese by Shemesh, Y. The Cronbach's α coefficient was 0.70, and the retest reliability was 0.96 (18).

### Health Belief Model

The researcher developed this study questionnaire based on the Rosenstock's Health Belief Model, which has been validated in Chinese hypertension patients (19). It contains the following four aspects: perceived susceptibility regarding self-awareness of infection and medication adverse reaction (three items); perceived seriousness regarding individual awareness of impact of rejection, infection and other complications and their survival (four items); perceived benefits of adherence to treatment with IM regarding subjective beliefs about whether better adherence lowers the possibility of complications (four items); and perceived barriers to adherence regarding the adverse effects of medication and some living conflicts (four items). Each item on the immunosuppressive medication belief questionnaire was structured using a 5-point Likert scale ranging from 1 (strongly disagree) to 5 (strongly agree). For the perceived barriers, the scaling was the opposite of that of the other constructs. The reliability of each questionnaire was tested using Cronbach's α. The range of Cronbach's α among Chinese patients was 0.77–0.90.

### Theory of Planned Behavior

We adapted the TPB questionnaire initially developed and validated in kidney transplant patients by Chisholm et al. (20). The Chinese version of the TPB has been validated in Chinese kidney transplant patients (21). The questionnaire explored attitudes (twelve items), perceived behavioral control (two items), subjective norms (five items), intentions (two items) and past behavior (two items). Evidence for the reliability and predictive validity of the TPB model has been provided by numerous studies (22, 23). The Cronbach's α of each variable was 0.87, 0.86, 0.76, 0.83, and 0.82 (8).

### Beliefs About Medication Questionnaire

The BMQ was used to evaluate the IM beliefs of renal transplant patients. It was developed by Horne regarding medicine use by patients with chronic diseases, such as the qualitative interview summary of belief, which has been widely used abroad (24). The scale was translated into Chinese in 2013 by Lv and was used to evaluate medication non-adherence among elderly patients with depressive disorder. The scale consists of four subscales (specific necessity, specific concerns, general harm and general overuse), for a total of 18 items. All of the items are scored on a 5-point Likert scale from “very inconsistent” to “very consistent”, and medication belief is calculated as the difference between the specific necessity and specific concerns scales, with a range of −20 to +20. A positive score indicates that the patients rated their beliefs in the necessity of taking medications higher than concerns about the medication and vice versa. The Cronbach's α coefficient of the scale was 0.65 (25).

### Perceived Social Support Scale

Perceived social support was measured by the PSSS, which was validated in the Chinese context by Li et al. (26) and Zimet et al. (27). The PSSS is a 12-item self-report scale that assesses perceived support arising from three dimensions, namely family support (e.g., “I get the emotional help and support I need from my family”), friend support (e.g., “I can count on my friends when things go wrong”), and others support (e.g., “There is a special person in my life who cares about my feelings”). Each item is scored on a seven-point scale ranging from 1 (completely disagree) to 7 (completely agree). Total scores can range from 12 to 84, with higher scores indicating greater perceived social support. Cronbach's α of the PSSS was 0.921 (28).

### General Self-Efficacy Scale

The GSES was originally developed by Schwarzer and Aristi and has mainly been used to measure confidence in the face of difficulties and setbacks (29). The Chinese version of the GSES was developed by Wang et al. (30). The 10-item scale only includes one dimension, and each item is scored on a 4-point Likert scale from 1 (not true at all) to 4 (exactly true). The GSES score is the sum of all of the items divided by the number of items, and the higher that the score is, the stronger that self-efficacy is. The Cronbach's α of the scale was 0.87, the retest reliability was 0.83, and the half reliability was 0.90 (30).

### ML Model Building

A total of five ML models were developed in our study, including logistic regression (LR), random forest (RF), multilayer perceptron (MLP), support vector machine (SVM) and eXtreme Gradient Boosting (XGBoost). The parameters from the combined theory, which was introduced as above, were used as independent variables to predict immunosuppression non-adherence in kidney transplant recipients. The synthetic minority oversampling technique (SMOTE) method was adopted to solve the problem of unbalanced classification of patient data samples. *K*-fold cross validation (*k* = 5) was adopted to find the optimal hyperparameters. After five rounds of training/validation rotation, the average sensitivity, specificity, accuracy, positive predictive value (PPV), negative predictive value (NPV) and area under the curve (AUC) were calculated to compare the performance of different ML models. All of the ML models were built with Python software, version 3.6, and the ML library scikit-learn. The SHapley Additive exPlanations (SHAP) method was used to evaluate the contribution of predictors to predicting the risk of immunosuppressant medication non-adherence in RTPs.

### Statistical Analysis

The sociodemographic characteristics were analyzed using descriptive statistics. Categorical variables were summarized using numbers or percentages. Statistical analysis was performed using SPSS software, version 20.0 (SPSS, Inc., Chicago, Ill, United States).

## Results

### Characteristics of the Participants

In the training model, a total of 1,237 questionnaires were distributed, of which 1,011 were analyzed, yielding a response rate of 81.7%. The distribution of the general data is shown in Table 1.

**Table 1:** Patient characteristics (*N* = 1,011).

**Characteristics**		***N* (%)**	**Characteristics**		***N* (%)**
Age (y)	18–20	18 (1.8)	Household income (RMB)	≤ 3,000	380 (37.6)
	21–30	116 (11.5)		3,000–5,000	329 (32.5)
	31–40	343 (33.9)		>5,000	302 (29.9)
	41–50	336 (33.2)	Time after transplantation (month)	≤ 6	102 (10.1)
	≥ 51	198 (19.6)		6–12	120 (11.9)
Sex	Male	569 (56.3)		12–36	355 (35.1)
	Female	442 (43.7)		≥36	434 (42.9)
BMI	<18.5	154 (15.2)	Organ source	DCD	870 (86.1)
	18.5–24	610 (60.3)		Relative donor	141 (13.9)
	24–28	207 (20.5)	Drug side effects before transplantation	No	541 (53.5)
	>28	40 (4.0)		Yes	470 (46.5)
Work	Yes	430 (42.5)	Preoperative medication reminder method	No	126 (12.5)
	No	581 (57.5)		Yes	885 (87.5)
Education	≤ Secondary school	266 (26.3)	Use pill box before transplantation	No	558 (55.2)
	High school	344 (34.0)		Yes	453 (44.8)
	College degree or above	401 (39.7)	Drug side effects after transplantation	No	319 (31.6)
Marital status	Unmarried	188 (18.6)		Yes	692 (68.4)
	Married	719 (71.1)	Postoperative medication reminder method	No	62 (6.1)
	Divorced/Widowed	104 (10.3)		Yes	949 (93.9)
Religion	No	933 (92.3)	Use pill box after transplantation	No	383 (37.9)
	Yes	78 (7.7)		Yes	628 (62.1)
Preoperative drinking history	No	596 (59.0)			
	Yes	415 (41.0)			

*RMB, Chinese Yuan Renminbi; DCD, Donation after Cardiac death. The DCD classification in our study was based on The Chinese classification standard, not the international classification standard*.

### The IM Non-adherence in the Participants

In general, 389 of the 1,011 participants (38.5%) were determined to have IM non-adherence over the last 4 weeks according to the BAASIS results, the details of which are shown in Table 2. Specifically, missing the prescribed medication time was the most common cause of IM non-adherence, which had the highest rate of 27.8%. Among patients, 14.9 and 9.7% missed the medication time 1 time and 2–3 times over the last 4 weeks, respectively. The second most common cause was missing one dose of IM, with a rate of 21.6%. 3.3% of the participants completely stopped the intake of IM without doctors' advice.

**Table 2:** Adherence to IM measured by BAASIS.

**Item number**		**No. (%)**
1A	Taking non-adherence: Yes/No	218 (21.6) / 793 (78.4)
	1 occasion	166 (16.4)
	2 or more occasions	52 (5.2)
1B	Drug-holidays: Yes / No	122 (12.1) / 889 (87.9)
	1 occasion	94 (9.3)
	2 or more occasions	28 (2.8)
2	Timing adherence: Yes/No	281 (27.8) / 730 (72.2)
	1 occasion	151 (14.9)
	2–3 occasions	98 (9.7)
	4–5 occasions	15 (1.5)
	Every 2–3 days	14 (1.4)
	Almost every day	3 (0.3)
3	Dose-alteration: Yes/No	62 (6.2) / 949 (93.8)
4	Discontinuation Yes/No	33 (3.3) / 978 (96.7)

### ML Models to Predict IM Non-adherence

A total of five ML models, including LR, RF, MLP, SVM, and XGBoost, were developed to predict IM non-adherence based on the variables of the combined theory. The method of recursive feature elimination (RFE), a feature selection algorithm to iteratively remove irrelevant features based on the model performance on the cross-validation result, was used to identify the most relevant features to build ML models. After screening, a total of 10 features were selected to build ML models: age, marital status, HBM-perceived barriers, use pill box after transplantation, PSSS- family support, drug side effects before transplantation, TPB attitudes, time after transplantation, household income and drug side effects after transplantation, the assignments of predictors showed in Table 3.

**Table 3:** Predictors' assignment of ML of RTPs' medication adherence.

**Predictors**	**Assignment**
Medication adherence	Adherence = 1; Non-adherence = 2
Age (y)	18–20 = 1; 21–30 = 2; 31–40 = 3; 41–50 = 4; ≥51 = 5
Marital status	Unmarried = 1; Married = 2; Divorced/Widowed = 3
Household income (Yuan)	≤ 3,000 = 1; 3,000–5,000 = 2; >5,000 = 3
Time after transplantation (m)	≤ 6 = 1; 6–12 = 2; 12–36 = 3; ≤ 36 = 4
Drug side effects before transplantation	None = 1; 1type = 2; 2types = 3; 3types = 4
Drug side effects after transplantation	None = 1; 1type = 2; 2types = 3; 3types = 4; 4types = 5; 5types = 6
Use pill box after transplantation	No = 1; Yes = 2
TBP-attitudes	Continuous value
PSSS-family support	Continuous value
HBM-perceived barriers	Continuous value

**Figure 2:**
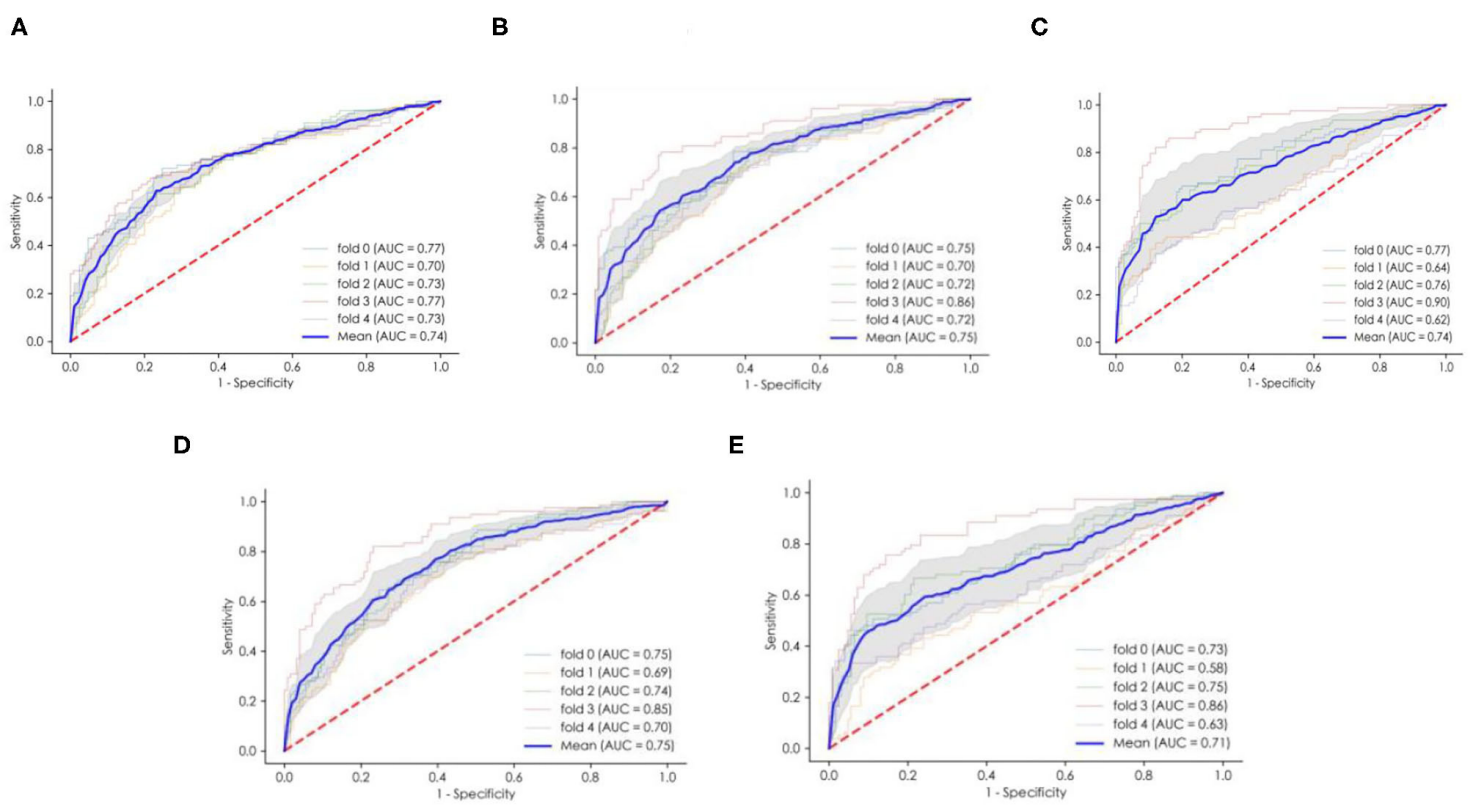
The ROC curves and average AUC of the ML models. **(A)** The logistic regression (LR) model. **(B)** The multilayer perceptron (MLP) model. **(C)** The random forest (RF) model. **(D)** The support vector machine (SVM) model. **(E)** eXtreme Gradient Boosting (XGBoost). ROC curve, receiver operating characteristic curve. AUC area under the curve.

The performance of the ML models is shown in Table 4. All of the models had good performance in predicting non-adherence, and their AUCs were all >0.70 (Figure 2). Among them, the SVM model had the greater AUC of 0.750.The formulation of the SVM model was as follows: Sign (−0.559006 ^*^age + −0.295894 ^*^Marital status + −0.277909 ^*^ HBM-perceived barriers + −0.073360^*^ Use pill box after transplantation + −0.272830 ^*^ PSSS-family support + 0.236412 ^*^Drug side effects before transplantation + −0.077296 ^*^TBP-attitudes + 0.202987^*^Time after transplantation + 0.192931^*^ Household income + 0.064712 ^*^Drug side effects after transplantation + −0.530045).

**Table 4:** The performance of the ML models in predicting IM non-adherence.

**Model**	**Sensitivity**	**Specificity**	**ppv**	**npv**	**Accuracy**	**roc_auc**
LR	0.66	0.72	0.60	0.77	0.69	0.742
SVM	0.59	0.73	0.58	0.75	0.68	0.750
MLP	0.63	0.73	0.59	0.76	0.69	0.749
RF	0.55	0.82	0.66	0.75	0.72	0.739
XGBoost	0.57	0.76	0.60	0.74	0.69	0.710

### Validation by External Data

We recruited 180 other patients as external data to validate the performance of the ML models. Detailed information about the patients is shown in the Supplementary Data. All of the ML models except for XGBoost had good prediction performance, with AUCs >0.6. The details of other models are shown in Table 5. The SVM model had the greater AUC of 0.668, and the sensitivity, specificity, PPV, NPV and accuracy were 0.62, 0.66, 0.53, 0.74 and 0.64, respectively.

**Table 5:** The performance of the ML models validated by the external data.

**Model**	**Sensitivity**	**Specificity**	**PPV**	**NPV**	**Accuracy**	**roc_auc**
LR	0.51	0.63	0.46	0.68	0.59	0.630
SVM	0.62	0.66	0.53	0.74	0.64	0.668
MLP	0.59	0.63	0.49	0.71	0.61	0.641
RF	0.43	0.78	0.54	0.69	0.64	0.636
XGBoost	0.57	0.47	0.40	0.65	0.51	0.552

### Further Explanation of the Prediction Model

**Figure 3:**
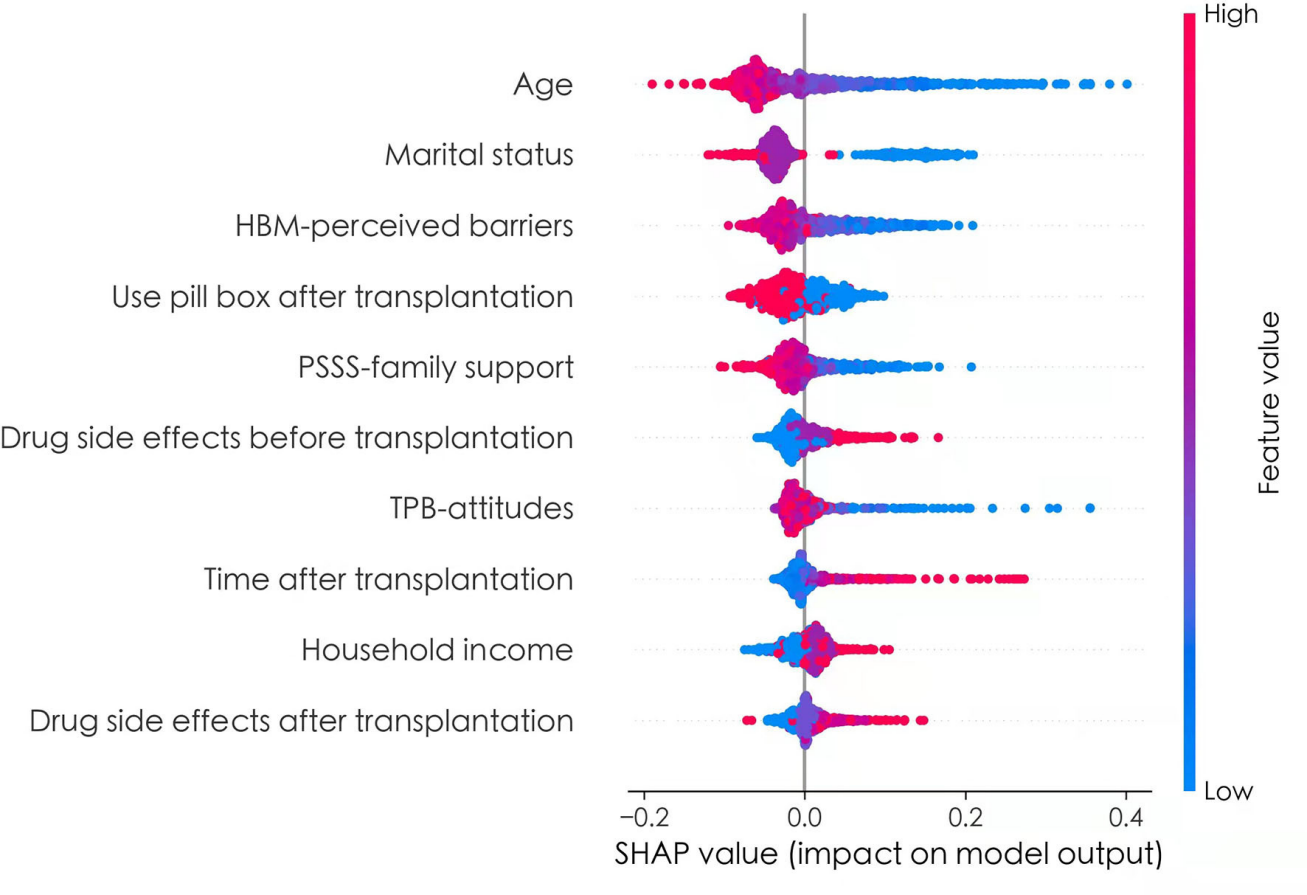
The SHAP values of predictors.

To better explain the effects of predictors of the prediction model, the SHAP method was used to evaluate the importance of predictors. The application of SHAP method was based on derivation set. The Figure 3 showed the SHAP values for each feature plotted for each sample. Each line represented a feature, and the abscissa is the SHAP value. A dot represented a sample, and the ordinate represented feature value (red is high, blue is low). From Figure 3, we can see that age, marital status, HBM-perceived barriers, use pill box after transplantation, PSSS-family support, and TPB-Attitudes had negative effects on predicting the risk of medication non-adherence, and drug side effects before transplantation, time after transplantation, household income, and drug side effects after transplantation exerted positive effects on predicting the risk of medication non-adherence.

**Figure 4:**
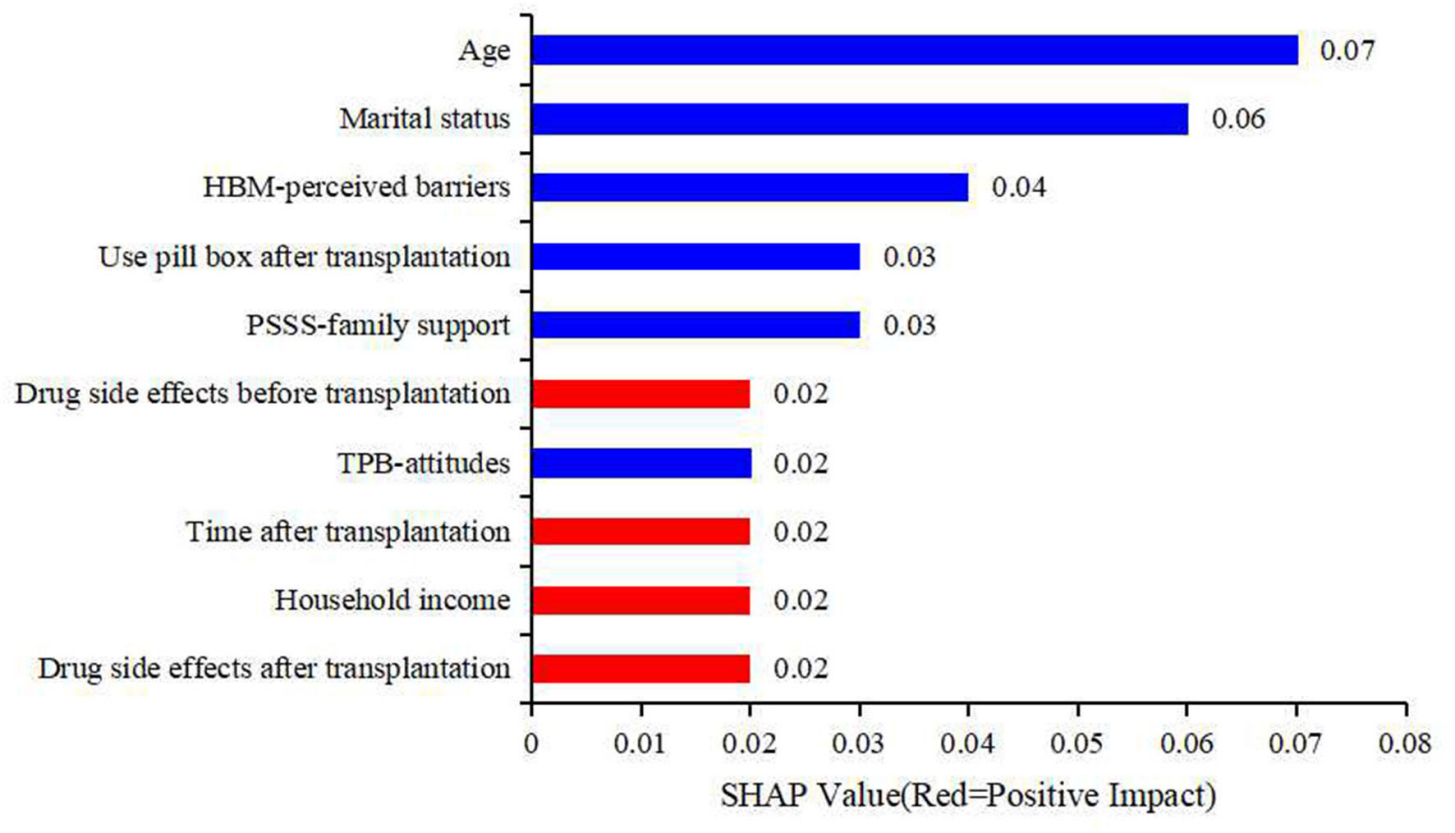
The rank of the SHAP values of predictors.

To make it easier to understand, SHAP provided another way to calculate the importance of features, that was to take the average value of the absolute value of SHAP value of each feature as the importance of the feature, and get a standard bar graph (Figure 4). The blue bar was the negative influence and the red bar was the positive influence (It needs to be explained here that the meaning of positive influence and negative influence depends on our assignment, and the specific assignment is shown in Table 3). In Figure 4, the top five factors predicting medication non-adherence were age, marital status, HBM-perceived barriers, use pill box after transplantation, and PSSS-family support, with the mean values of 0.07, 0.06, 0.04, 0.03, and 0.03, respectively.

## Discussion

Renal transplantation is the most effective treatment for end-stage renal disease. IM non-adherence is one of the main reasons for transplant renal failure (7, 31). Medicine taking among transplant recipients is a complex and ubiquitous task with significant impacts on outcomes (32). Among solid organ transplant patients, renal transplant patients had the highest IM non-adherence, ranging from 20 to 70% (6, 17, 33–36). In this study, the IM non-adherence of the patients was 38.5%, and taking IM more than 2 h before or after the recommended dosing time in the last 4 weeks was the most common issure of IM non-adherence, which occurred at a rate of 27.8%. This result was similar to the results of our previous study (5), which also suggested that the problem of medication non-adherence in RTPs remains serious, especially not taking medication on time. Therefore, there is an urgent need to predict the risk factors for IM non-adherence and to undertake targeted intervention methods to improve the situation. Targeting of patients who are at risk for non-adherence to IM and provide them with focused interventions could help to improve kidney transplant outcomes in this high-risk group.

In recent years, numbers of studies have been published discussing the factors that influence medication non-adherence among RTPs. Two systematic reviews summarized the risk factors associated with medication non-adherence in RTPs, including five categories: social and economic factors; therapy-related factors; patient-related factors; condition-related factors; health care team and system-related factors (7, 37). In our study, a total of 10 features (age, marriage status, HBM-perceived barriers, and so on) were screened out and included in the classification. Although the factors have been discussed, many studies still failed to evaluate how risk factors can be utilized to predict the risk of non-adherence behavior. A study conducted in 99 US-based transplant centers showed that 71.1% of health care professionals acknowledged not having a prospective medication non-adherence screening protocol, and little is known about strategies currently utilized by transplant centers to monitor or manage this issue (38). To solve this common problem, some scholars have attempted to establish predictive models of non-adherence in patients after kidney transplantation, such as HBM and TPB models (5, 8, 20, 39). However, models currently constructed has several limitations, such as insufficient coverage variables, inability to quantify risk prediction, inability to be directly applied to clinical practice, and only providing theoretical reference value. Therefore, it is necessary to establish a more ideal prediction model of drug disobedience in patients after kidney transplantation with the help of stronger calculation methods, according to the requirements of the prediction model. Our study aimed to assess multiple ML technologies and screen out a model that could be better used to predict patient non-adherence risks.

Compared with models in previous studies, the model constructed in this study had better performance in four aspects.

(1) Methodology. ML methods have advantages in addressing non-linear relationships between many risk factors and outcomes compared to traditional logistic regression analysis. Based on machine learning technology, five models were established, and the models were evaluated and compared. The final results showed that the SVM model best predicted IM use among RTPs, and the calculation equation was given. SVM aims at finding the best hyperplane to divide feature spaces for different categories with the maximum margin. It can be used for classification, regression and outlier detection. The model is efficient in high-dimensional spaces and robust against imbalances of categories. (2) Data processing. In the data preprocessing stage, we adopted SMOTE to augment the positive samples and create new synthetic patients with medicine non-adherence to balance the training dataset and increase it variety and to improve the prediction accuracy using positive samples. In the factor screening process, we adopted recursive feature elimination (RFE), a feature selection algorithm, to iteratively remove irrelevant features from the dataset based on the model performance of the cross-validation result. Finally, we selected the most relevant features on average for each model. (3) Practicality. The SVM model yielded the calculation equation, and the specific risk prediction value could be calculated through the input variables to quantify the risk prediction. To better reflect this model, we used the SHAP method to rank the importance of risk factors. According to the SHAP value, the main factors affecting the medication adherence of renal transplant recipients were age and marital status, followed by HBM-perceived barriers, use pill boxes after transplantation, and family support.

From the model, the younger that the age was, the higher that the risk of non-adherence was. Many studies have suggested that younger age is a risk factor for non-adherence (37, 40, 41). Older patients face issues, such as comorbidities, physical limitations and social isolation, that can lead to two contradictory outcomes: non-adherence or better awareness of their limits and closer attention paid to drug regimens and medical follow-up (41). Ladin et al. indicated that marital status was associated with clinical outcomes (42). Our study revealed that unmarried recipients were at higher risk of medication non-adherence after transplantation, which might have been due to the lack of family member-based supervision. In our study, lower levels of perceived barriers to taking IM remained significant predictors of better IM adherence, consistent with our previous study (6). RTPs who did not use a pill box were at higher risk of non-adherence, which could be lead to medication omission and incorrect dosage. Through a literature review, we found that this factor rarely appeared in other studies. Renal transplant patients often need to take multiple drugs in different ways. The design of the pill box considers the functions of portability and separation. It can remind patients to take medicine on time with the correct dosage and help them to establish a good habit of taking medicine to improve their medication adherence (43). The patients with lower family support were at greater risk of non-adherence. Emotional support and daily support provided by family members could reduce the incidence of forgetfulness and improve IM among RTPs. Chisholm-Burns et al. noted a positive association between greater family support and adherence, consistent with our findings (44).

The established prediction model must be applied in clinical practice to realize application value. Our results could help medical institutions to predict the risk of non-adherence among RTPs and provide direction for the development of appropriate interventions. On the one hand, risk factors for non-adherence can also be divided into modifiable and non-modifiable risk factors in our study, which are of key importance when attempting to resolve non-adherence. For non-modifiable factors, such as patient-related factors (age, marriage status, household income), we can target patients with these characteristics and monitor medication adherence earlier. Modifiable risk factors, such as HBM-perceived barriers and TPB attitudes, can be modified by providing education about the need for such medications and medication-taking self-efficacy. On the other hand, we could develop an online evaluation tool based on the SVM model with the best prediction effect and apply the ML predictive model to practice in the future. It allows patients and doctors to use it anytime and anywhere, with a friendly interface. No registration or login password is required, which could greatly relieve doctors' work pressure and strained medical resources. For patients with a higher predicted risk, interventions can be introduced to reduce the risk in advance.

## Conclusion and Implications

In summary, it was necessary to improve the IM adherence of RTPs. Ten risk predictors, such as age and marital status, were screened to predict the risk of IM among RTPs. Through ML, we built an SVM model that could better predict the risk of IM non-adherence, which could guide our clinical practice and help us to quickly identify high-risk patients. For modifiable risk predictors, future studies could undertake corresponding intervention measures to reduce the incidence of non-adherence. Unmodifiable risk predictors could help us to identify risk groups earlier and undertake interventions early.

## Limitations

The highlights of the study include the processing of a larger sample size, screening for risk factors and building of a prediction model based on ML. Admittedly, the current study still has some limitations that should be considered as follows. (1) The sample size calculation was absent, which might have led to systematic errors. System error, it is a kind of random error, as a result of sample is non-random. As our data from six transplant centers, distribution in the two cities, sample source areas and places are based on the available resources, rather than random access, although large sample data can reduce the system error, but is unable to avoid. (2) The questionnaires used in our study were self-reported, and the results might have been affected by the patients' subjective judgment. (3) Randomization was not employed in the selection of participants, and selection bias might have impacted the outcomes of this study.

## Data Availability Statement

The raw data supporting the conclusions of this article will be made available by the authors upon reasonable request. Requests to access these datasets should be directed to Jia Liu, chucklejl@163.com.

## Ethics Statement

The studies involving human participants were reviewed and approved by Ethics Committee of The Third Xiangya Hospital (2019-SS161), Changsha, China. The patients/participants provided their written informed consent to participate in this study.

## Author Contributions

XZ: investigation, data curation, and writing—original draft. BP: software, investigation, and resources. QY: software and methodology. JL: conceptualization, supervision, and methodology. JY: writing—review and editing and visualization. All authors contributed to the article and approved the submitted version.

## Funding

This work was partly supported by the National Natural Science Foundation of China (No. 71904209).

## Conflict of Interest

The authors declare that the research was conducted in the absence of any commercial or financial relationships that could be construed as a potential conflict of interest.

## Publisher's Note

All claims expressed in this article are solely those of the authors and do not necessarily represent those of their affiliated organizations, or those of the publisher, the editors and the reviewers. Any product that may be evaluated in this article, or claim that may be made by its manufacturer, is not guaranteed or endorsed by the publisher.

## References

[1] Weng L Yang Y Huang H Chiang Y Tsai Y. Factors that determine self-reported immunosuppressant adherence in kidney transplant recipients: a correlational study. J Adv Nurs. (2017) 73:228–39. 10.1111/jan.13106 27532342

[2] Ganjali R Sabbagh MG Nazemiyan F. Factors associated with adherence to immunosuppressive therapy and barriers in Asian Kidney Transplant Recipients (vol 8, pg 53, 2019). Immunotargets Therapy. (2020) 9:141. 10.2147/ITT.S258315 31807474 PMC6844196

[3] Griva K Neo HLM Vathsala A. Unintentional and intentional non-adherence to immunosuppressive medications in renal transplant recipients. Int J Clin Pharm-Net. (2018) 40:1234–41. 10.1007/s11096-018-0652-6 29872960

[4] Liu J Zhu X Yan J Gong L Wu X Liu M. Association between regulatory emotional self-efficacy and immunosuppressive medication adherence in renal transplant recipients: does medication belief act as a mediator? Front Pharmacol. (2021) 12:559368. 10.3389/fphar.2021.559368 33762931 PMC7982474

[5] Zhang P Zhu X Yan J Liu J. Identification of immunosuppressive medication nonadherence factors through a combined theory model in renal transplant recipients 6-12. Front Pharmacol. (2021) 12:655836. 10.3389/fphar.2021.655836 34122077 PMC8187913

[6] Xia M Yan J Liu S Liu J. Beliefs of immunosuppressive medication among chinese renal transplant recipients, as assessed in a cross-sectional study with the basel assessment of adherence to immunosuppressive medications scale. Transpl Proc. (2019) 51:742–8. 10.1016/j.transproceed.2018.10.029 30979459

[7] Rebafka A. Medication adherence after renal transplantation-a review of the literature. J Renal Care. (2016) 42:239–56. 10.1111/jorc.12181 27629770

[8] Teng S. Using the Theory of Planned Theory (TPB) to Investigate the Factors of Immunosuppressive Medication Adherence Among Liver Transplant Recipients. Beijing: Beijing University of Chinese Medicine (2016). p. 71.

[9] Landolfi A Ricciardi C Donisi L Cesarelli G Troisi J Vitale C. Machine learning approaches in Parkinson's disease. Curr Med Chem. (2021) 28:6548–68. 10.2174/0929867328999210111211420 33430721

[10] Wang D Li J Sun Y Ding X Zhang X Liu S. A machine learning model for accurate prediction of sepsis in ICU patients. Front Public Health. (2021) 9:754348. 10.3389/fpubh.2021.754348 34722452 PMC8553999

[11] Beam AL Kohane IS. Big data and machine learning in health care. J Am Med Assoc. (2018) 319:1317–8. 10.1001/jama.2017.18391 29532063

[12] Tang J Liu R Zhang Y Liu M Hu Y Shao M. Application of machine-learning models to predict tacrolimus stable dose in renal transplant recipients. Sci Rep. (2017) 7:42192. 10.1038/srep42192 28176850 PMC5296901

[13] Feng Y. Prediction Model of Postoperative Recurrence of Early Non-small Cell Lung Cancer Based on Machine Learning. Shanghai: Shanghai University of Finance and Economics (2020). p. 73.

[14] Chen T Li X Li Y Xia E Qin Y Liang S. Prediction and risk stratification of kidney outcomes in IgA nephropathy. Am J Kidney Dis. (2019) 74:300–9. 10.1053/j.ajkd.2019.02.016 31031086

[15] Segal Z Kalifa D Radinsky K Ehrenberg B Elad G Maor G. Machine learning algorithm for early detection of end-stage renal disease. BMC Nephrol. (2020) 21:1–10. 10.1186/s12882-020-02093-0 33246427 PMC7693522

[16] Yoo KD Noh J Lee H Kim DK Lim CS Kim YH. A machine learning approach using survival statistics to predict graft survival in kidney transplant recipients: a multicenter cohort study. Sci Rep. (2017) 7:1–12. 10.1038/s41598-017-08008-8 28827646 PMC5567098

[17] Liu J Liu S Yan J Yi Q Huang H. Adherence to immunosuppressive medication in renal transplant recipients from follow-up outpatient in China: association of 2 different measurement methods. Clin Ther. (2015) 37:2572–80. 10.1016/j.clinthera.2015.09.014 26519232

[18] Shang YB Teng S Liu HX Wang L Zhang J Lin XH. Reliability and validity of Chinese version of Basel assessment of adherence to immunosuppressive medication scale in accessing liver transplant recipients. J Nurs Admin. (2017) 17:17–9.

[19] Zhao Y Chen L Qi W Wang B. Application of the health belief model to improve the understanding of antihypertensive medication adherence among Chinese patients. Patient Educ Couns. (2015) 98:669–73. 10.1016/j.pec.2015.02.007 25746128

[20] Chisholm MA Williamson GM Lance CE Mulloy LL. Predicting adherence to immunosuppressant therapy: a prospective analysis of the theory of planned behaviour. Nephrol Dial Transpl. (2007) 22:2339–48. 10.1093/ndt/gfm149 17442741

[21] Du C Wu S Liu H Hu Y Li J. Influencing factors of the intention of taking medicine among renal transplant recipients. Nurs Sci. (2018) 33:33–5.

[22] Andrew BJ Mullan BA de Wit JBF Monds LA Todd J Kothe EJ. Does the theory of planned behaviour explain condom use behaviour among men who have sex with men? A meta-analytic review of the literature. Aids Behav. (2016) 20:2834–44. 10.1007/s10461-016-1314-0 26860535

[23] Caru M Curnier D Levesque A Sultan S Marcil V Laverdiere C. Children's physical activity behavior following a supervised physical activity program in pediatric oncology. J Cancer Res Clin. (2020) 146:3037–48. 10.1007/s00432-020-03294-8 32583234 PMC11804408

[24] Horne R Weinman J. Patients' beliefs about prescribed medicines and their role in adherence to treatment in chronic physical illness. J Psychosom Res. (1999) 47:555–67. 10.1016/S0022-3999(99)00057-4 10661603

[25] Lv Y Li Z Han MY Xianhui M Cheng G An FR. The reliability and validity of the Chinese version of Beliefs about Medical Questionnaire among elderly patients with depressive disorder. Chin J Nursing. (2014) 49:389–93. 10.3761/j.jssn.0254.1769.2014.04.001

[26] Huang L Jiang Qj Ren WH. Coping, perceived social support and psychosomatic symptom among cancer patients. Chin Mental Health J. (1996) 10:160–1.7171884

[27] Zimet GD Powell SS Farley GK Werkman S Berkoff KA. Psychometric characteristics of the multidimensional scale of perceived social support. J Person Assess. (1990) 55:610–7. 10.1207/s15327752jpa5503&amp;4_17 2280326

[28] Huo X. Study on the Relationship Between the Health Literacy, Perceived Social Support and Quality of Life of Patients With Coronary Heart Disease. Nanchang: Nanchang University (2018), p. 73.

[29] Schwarzer R Aristi B. Optimistic self-beliefs :Assessment of general perceived self efficacy in thirteen cultures. Word Psychol. (1997) 3:177–90.

[30] Wang CK Hu ZF Liu Y. Reliability and validity of general self-efficacy scale. Chin J Appl Paychol. (2001) 37–40. doi: 1006-6020(2001)-01-0037-04

[31] Prihodova L Nagyova I Rosenberger J Majernikova M Roland R Groothoff JW. Adherence in patients in the first year after kidney transplantation and its impact on graft loss and mortality: a cross-sectional and prospective study. J Adv Nurs. (2014) 70:2871–83. 10.1111/jan.12447 24853863

[32] Tang J Kerklaan J Wong G Howell M Scholes-Robertson N Guha C. Perspectives of solid organ transplant recipients on medicine-taking: systematic review of qualitative studies. Am J Transplant. (2021) 21:3369–87. 10.1111/ajt.16613 33866675

[33] Mellon L Doyle F Hickey A Ward KD De Freitas DG Mccormick PA. Interventions for Improving Medication Adherence in Solid Organ Transplant Recipients. (2017). Available online at: https://www.ncbi.nlm.nih.gov/pmc/articles/PMC6486115/ (accessed August 14, 2021).

[34] Weng FL Chandwani S Kurtyka KM Zacker C Chisholm-Burns MA Demissie K. Prevalence and correlates of medication non-adherence among kidney transplant recipients more than 6 months post-transplant: a cross-sectional study. BMC Nephrol. (2013) 14:261. 10.1186/1471-2369-14-261 24289809 PMC4219432

[35] Reese PP Bloom RD Trofe-Clark J Mussell A Leidy D Levsky S. Automated reminders and physician notification to promote immunosuppression adherence among kidney transplant recipients: a randomized trial. Am J Kidney Dis. (2017) 69:400–9. 10.1053/j.ajkd.2016.10.017 27940063

[36] Paterson TSE O'Rourke N Shapiro RJ Thornton WL. Medication adherence in renal transplant recipients: a latent variable model of psychosocial and neurocognitive predictors. PLoS ONE. (2018) 13:e02042199. 10.1371/journal.pone.0204219 30265697 PMC6161882

[37] Belaiche S Decaudin B Dharancy S Noel C Odou P Hazzan M. Factors relevant to medication non-adherence in kidney transplant: a systematic review. Int J Clin Pharm Net. (2017) 39:582–93. 10.1007/s11096-017-0436-4 28374343

[38] Patel SJ Hofmeyer BA Moore CA Descourouez JL Nguyen DT Graviss EA. Medication nonadherence monitoring management in adult kidney transplantation: a survey of practices perceptions at US-based transplant programs. J Am Coll Clin Pharm. (2021) 4:1100–108. 10.1002/jac5.147725855820

[39] Kung PC Yeh MC Lai MK Liu HE. Renal transplant recipients: the factors related to immunosuppressive medication adherence based on the health belief model. J Nurs Res. (2017) 25:392–7. 10.1097/JNR.0000000000000181 28877127

[40] Gokoel SR Gombert-Handoko KB Zwart TC van der Boog PJ Moes DJ de Fijter JW. Medication non-adherence after kidney transplantation: a critical appraisal and systematic review. Transplant Rev Orlan. (2020) 34:1–18. 10.1016/j.trre.2019.100511 31627978

[41] Spivey CA Chisholm-Burns MA Damadzadeh B Billheimer D. Determining the effect of immunosuppressant adherence on graft failure risk among renal transplant recipients. Clin Transplant. (2014) 28:96–104. 10.1111/ctr.12283 24329814

[42] Ladin K Daniels A Osani M Bannuru RR. Is social support associated with post-transplant medication adherence and outcomes? A systematic review and meta-analysis. Transplant Rev Orlan. (2018) 32:16–28. 10.1016/j.trre.2017.04.001 28495070 PMC5658266

[43] Peng A. Effect of family medication box on medication compliance and prognosis of stroke patients. Nurs Pract Res. (2016) 13:39–40. 10.3969/j.issn.1672-9676.2016.08.017

[44] Chisholm-Burns MA Spivey CA Wilks SE. Social support and immunosuppressant therapy adherence among adult renal transplant recipients. Clin Transplant. (2010) 24:312–20. 10.1111/j.1399-0012.2009.01060.x 19694770

