# Corrigendum: Prediction model of immunosuppressive medication non-adherence for renal transplant patients based on machine learning technology

**DOI:** 10.3389/fmed.2022.964157

**Published:** 2022-08-09

**Authors:** Xiao Zhu, Bo Peng, QiFeng Yi, Jia Liu, Jin Yan

**Affiliations:** ^1^Nursing School of Central South University, Changsha, China; ^2^Nursing Department of Third Xiangya Hospital of Central South University, Changsha, China; ^3^Research Center of Chinese Health Ministry on Transplantation Medicine Engineering and Technology, The Third Xiangya Hospital, Central South University, Changsha, China

**Keywords:** immunosuppressive medication, non-adherence, prediction model, renal transplant patients, machine learning technology

In our published article, there was an error in Table 2 as published. Table 2 used a scale Basel Assessment of Adherence to Immunosuppressive Medications Scale (BAASIS), which was authorized by the original developer Dr. De Geest. Dr. De Geest contacted us recently. He suggested that the Table 2 should be presented like their team. Therefore, we would like to replace Table 2. The corrected Table 2 and its caption appear below.

**Table 2:** Adherence to IM measured by BAASIS.

**Item number**		**No. (%)**
1A	Taking non-adherence: Yes/No	218 (21.6) / 793 (78.4)
	1 occasion	166 (16.4)
	2 or more occasions	52 (5.2)
1B	Drug-holidays: Yes / No	122 (12.1) / 889 (87.9)
	1 occasion	94 (9.3)
	2 or more occasions	28 (2.8)
2	Timing adherence: Yes/No	281 (27.8) / 730 (72.2)
	1 occasion	151 (14.9)
	2–3 occasions	98 (9.7)
	4–5 occasions	15 (1.5)
	Every 2–3 days	14 (1.4)
	Almost every day	3 (0.3)
3	Dose-alteration: Yes/No	62 (6.2) / 949 (93.8)
4	Discontinuation Yes/No	33 (3.3) / 978 (96.7)

The authors apologize for this error and state that this does not change the scientific conclusions of the article in any way. The original article has been updated.

## Publisher's note

All claims expressed in this article are solely those of the authors and do not necessarily represent those of their affiliated organizations, or those of the publisher, the editors and the reviewers. Any product that may be evaluated in this article, or claim that may be made by its manufacturer, is not guaranteed or endorsed by the publisher.

