# Supplementary material for: Prediction Model of Immunosuppressive Medication Non-adherence for Renal Transplant Patients Based on Machine Learning Technology
Source: Front Med (Lausanne). 2022 Feb 18;9:796424. doi: 10.3389/fmed.2022.796424 (PMC8895304; doi:10.3389/fmed.2022.796424)
Supplement: Supplementary file 1 [file Data_Sheet_1.pdf]

Patient characteristics of external validation (N=180)

Characteristics		N(%)	Characteristics		N(%)
**Age(y)**	18-20	6(3.3)	**Household income（RMB）**	≦3000	67(37.2)
	21-30	18(10.0)		3000–5000	58(32.2)
	31-40	57(31.7)		>5000	55(30.6)
	41-50	61(33.9)	**Time after transplantation(month)**	≤6	35(19.5)
	≧51	38(21.1)		6–12	32(17.8)
**Sex**	Male	123(68.3)		12–36	53(29.4)
	Female	57(31.7)		≥36	60(33.3)
**BMI**	<18.5	28(15.6)	**Organ source**	DCD	161(89.4)
	18.5-24	105(60.3)		Relative donor	19(10.6)
	24-28	39(21.7)	**Drug side effects before transplantation**	No	80(44.4)
	>28	8(4.4)		Yes	100(55.6)
**Work**	Yes	87(48.3)	**Preoperative medication reminder method**	No	21(11.7)
	No	93(51.7)		Yes	159(88.3)
**Education**	≦Secondary school	45(25.0)	**Use pill box before transplantation**	No	118(65.6)
	High school	57(31.7)		Yes	62(34.4)
	College degree or above	78(43.3)	**Drug side effects after transplantation**	No	46(25.6)
**Marital status**	Unmarried	29(16.1)		Yes	134(74.4)
	Married	134(74.4)	**Postoperative medication reminder method**	No	2(1.1)
	Divorced/Widowed	17(9.5)		Yes	178(98.9)
**Religion**	No	163(90.6)	**Use pill box after transplantation**	No	41(22.8)
	Yes	17(9.4)		Yes	139(77.2)
**Preoperative drinking history**	No	101(56.1)			
	Yes	79(43.9)			
